# High Natality Rates of Endangered Steller Sea Lions in Kenai Fjords, Alaska and Perceptions of Population Status in the Gulf of Alaska

**DOI:** 10.1371/journal.pone.0010076

**Published:** 2010-04-08

**Authors:** John M. Maniscalco, Alan M. Springer, Pamela Parker

**Affiliations:** 1 Department of Science, Alaska SeaLife Center, Seward, Alaska, United States of America; 2 Institute of Marine Science, University of Alaska Fairbanks, Fairbanks, Alaska, United States of America; Institut Pluridisciplinaire Hubert Curien, France

## Abstract

Steller sea lions experienced a dramatic population collapse of more than 80% in the late 1970s through the 1990s across their western range in Alaska. One of several competing hypotheses about the cause holds that reduced female reproductive rates (natality) substantively contributed to the decline and continue to limit recovery in the Gulf of Alaska despite the fact that there have been very few attempts to directly measure natality in this species. We conducted a longitudinal study of natality among individual Steller sea lions (n = 151) at a rookery and nearby haulouts in Kenai Fjords, Gulf of Alaska during 2003–2009. Multi-state models were built and tested in Program MARK to estimate survival, resighting, and state transition probabilities dependent on whether or not a female gave birth in the previous year. The models that most closely fit the data suggested that females which gave birth had a higher probability of surviving and giving birth in the following year compared to females that did not give birth, indicating some females are more fit than others. Natality, estimated at 69%, was similar to natality for Steller sea lions in the Gulf of Alaska prior to their decline (67%) and much greater than the published estimate for the 2000s (43%) which was hypothesized from an inferential population dynamic model. Reasons for the disparity are discussed, and could be resolved by additional longitudinal estimates of natality at this and other rookeries over changing ocean climate regimes. Such estimates would provide an appropriate assessment of a key parameter of population dynamics in this endangered species which has heretofore been lacking. Without support for depressed natality as the explanation for a lack of recovery of Steller sea lions in the Gulf of Alaska, alternative hypotheses must be more seriously considered.

## Introduction

Between the late 1970s and 2000, the western distinct population segment (WDPS) of Steller sea lions (*Eumetopias jubatus*) declined by more than 80% in the Aleutian Islands and Gulf of Alaska (GOA) [1] and was listed as endangered in 1997. The designation led to years of unprecedented federal funding for studies aimed at determining the cause(s) of the decline and the reason(s) for a lack of recovery [2], [3]. The impetus derived in major part from two related factors: 1) the importance of walleye pollock (*Theragra chalcogramma*) to the nutritional status of the animals—pollock is widely consumed by Steller sea lions, and 2) pollock is the target of the largest single-species commercial fishery in the world, with an exvessel value in the order of half a billion dollars U.S. Yet despite the massive monetary expenditures and scientific effort, no consensus of opinion has emerged about the cause of the decline. However, two general classes of hypotheses have been proposed: top-down forcing, primarily by predation [4], [5]; and bottom-up forcing through changes in prey resources due to climate change or competition with commercial fisheries [4], [6], [7].

**Figure 1:**
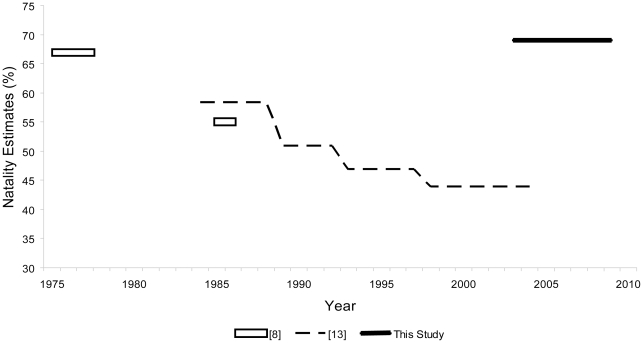
Estimates of Steller sea lion natality from 3 different studies spanning 4 decades.

The reproductive rate (natality—the number of young produced per reproductively mature female) of animals is an important life history characteristic and can be an indicator of nutritional status. In the context of bottom-up control of population dynamics, reduced natality of Steller sea lions [8] and low juvenile and adult survival [9], [10] due to poor nutrition are believed by some to have been the causes of the population collapse. Since 2000, some parts of the WDPS have experienced modest increases in abundance [11], [12]. Inferential population dynamic models based on census counts of Steller sea lions indicate that the recent small increases are related to improved juvenile and adult survival, but that natality continued to deteriorate during the 1990s and 2000s [10], [13]. Natality in the Central GOA was estimated to be 67% during the 1970s [14], 55% in the 1980s [8], and just 43% in the 2000s [13] (Figure 1).

Estimates of natality in the 1970s and 1980s were obtained by collecting females during early (October – November) and late (April – May) gestation and determining the proportion that were reproductively mature with a developing fetus [8]. Sources of error in natality calculations using those methods would have included variation in the status of females that were collected early and late, variation in abortion rates during the last month of gestation after late collections occurred, and potential violation of the assumption of random sample collection (e.g., bias towards collecting younger, more naïve, or bigger, more easily observed animals).

Now that sacrificing endangered Steller sea lions for science is no longer acceptable or permissible, broad-scale census counts of non-pups and pups, and estimates of the proportions of non-pups that are adult females and juveniles, have provided the primary data for estimating natality in the WDPS [10], [12], [13]. Those data and estimation procedures, however, may not be appropriate for an accurate assessment of natality when compared with earlier studies because they contain different sources of error, such as variations in the proportion of animals hauled out between censuses, the proportion of pups that have died and/or washed away prior to the censuses, the number of pups that have not yet been born at the time of the censuses, and proper determination of which animals are reproductively mature females. Furthermore, a shifting ocean climate [15], [16] may have caused systematic changes in sightability of these animals over time that led to an illusion of declining natality.

In this study, we emulated the earlier studies of natality in Steller sea lions [8], [14] without some of the potential biases by tracking known individuals over time (7 yrs). This obviated the need to estimate proportions of females hauled out on the rookery or the proportion of pups that had died prior to surveys, as need to be estimated from census counts, because both were fully accounted for by virtually continuous observations. Thus, the findings of our study are based on direct observations and are more directly comparable to the estimate of natality in the 1970s in the GOA, and they contrast with recently hypothesized estimates from an inferential model [13] in that they do not indicate a difference in natality between current levels and those in the 1970s. We will discuss the likely reasons for the incongruity in light of methodological considerations and changing ocean climate regimes, and how it affects our perception of the status of the population in the GOA and controls on their abundance.

## Results

One hundred and fifty one female Steller sea lions met the criteria for maturity and repeat sightability for at least two years. Females of known age (n = 6) gave birth for the first time at 5.3 yr (range: 4–6 yr).

**Figure 2:**
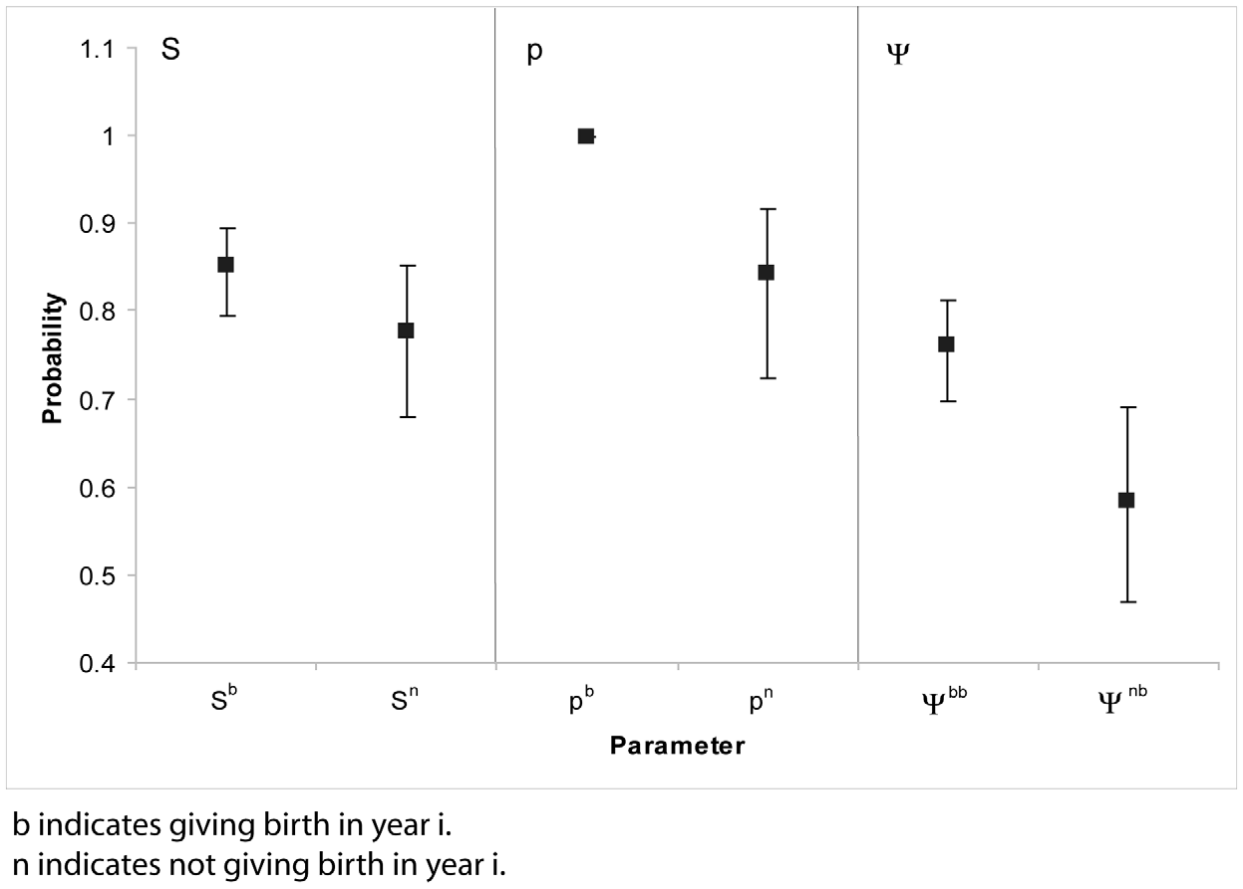
Survival (S), sighting probabilities (p), and state transitions (ψ) estimated from the most parsimonious model (S_st_ p_st_ ψ_st_).

Results of the GOF test indicated an insignificant degree of overdispersion to the data (ĉ = 1.10; χ^2^ = 51.67; d.f. = 47; *P* = 0.296). Nevertheless, to be conservative, the ĉ value was applied to tested models to inflate variances of estimated parameters. The most parsimonious model included both survival and state transitions as dependent on whether or not the female produced a pup in the previous year; however, the next best model, which did not include a difference in survival between states, was virtually identical (LRT: χ^2^ = 2.16; d.f. = 1; *P* = 0.142; Table 1). Together, the likelihood associated with these two models was 89%. Sighting probabilities were appropriately estimated at 0.999 for females giving birth and at 0.843 for those not giving birth (Figure 2).

**Table 1:** Kenai Fjords Steller sea lion multi-state mark-recapture models tested in Program MARK.

Model	#Par	QAICc	ΔQAICc	Weight	QDeviance
S_st_ p_st_ ψ_st_	6	698.062	0.00	0.456	235.592
S. p_st_ ψ_st_	5	698.151	0.09	0.436	237.751
S_t_ p_st_ ψ_st_	10	701.873	3.81	0.068	231.006
S_st.t_ p_st_ ψ_st_	16	703.552	5.49	0.029	219.715
S_st_ p_st_ ψ_st*t_	16	706.039	7.98	0.008	222.201
S_st_ p_st.t_ ψ_st_	16	708.604	10.54	0.002	224.767
S_st_ p_st_ ψ.	5	715.785	17.72	0.000	255.385
S_st_ p_st*t_ ψ_st*t_	26	719.762	21.70	0.000	213.254
S_st*t_ p_st*t_ ψ_st*t_	36	727.001	28.94	0.000	196.396

Survival (S), resight probability (p), and state transition (ψ) were tested for effects of state (st; B/N), year (t), or neither (.). All models tested were based on ĉ = 1.10.

Natality, estimated from results of the most parsimonious model, was 69.2% (±2.5%, SE; Figure 1) for all years combined and was fractionally higher when calculated from the next best model that expressed no difference in survival. Females giving birth had a higher probability of surviving to the following year (0.851) than females that did not give birth (0.777; Figure 2) but the nearly equivalent, second-best model indicated no difference in survival at 0.828 (±0.021). Also, females that gave birth in year *i* were more likely to give birth in year *i*+1 (ψ^bb^ = 0.760) than females that did not give birth in year *i* (ψ^nb^ = 0.584) with no overlap in confidence intervals (Figure 2). Results were similar from the second-best model indicating significant differences between these transitions.

## Discussion

Natality is not the only life history trait that can be influential in driving dynamics of populations and that is susceptible to effects of prey limitation under bottom-up forcing scenarios for pinnipeds in decline [17]–[19]. However, we made no measurements of other factors such as juvenile survival and recruitment. The focus of this study was on natality which is a critical element of special concern for Steller sea lions in Alaska.

Female Steller sea lions reach sexual maturity with their first ovulation at an average age of 4.6 y [14] and nearly all females that are mature become pregnant each year [8]. At Chiswell Island, known-age females (n = 6) produced their first pups at an average age of 5.3 y, indicating they were ovulating at 4.3 y, although we cannot necessarily assume that was their first ovulation. Yet, it is apparent from the data presented here that age at first reproduction was similar to that in the 1970s and justifies choosing females ≥5 years of age as part of this analysis for direct comparisons with earlier work on natality rates.

The estimate of natality found in this study (69%) was similar to natality in the 1970s (67%) [8], prior to the population decline in this region. However, our value may be slightly underestimated because of the possible inclusion of older, post-reproductive animals. There is some evidence that Steller sea lions become reproductively senescent at more than 20 years of age [14] and previously calculated natality for the 1970s did not include elderly, non-pregnant females because of potential biases [8]. At least two adult females of unknown age were included in our study and may have been post-reproductive, as they never gave birth over the 4+ years they were observed. Future studies of known-age individuals should help to clarify the extent of senescence in this species.

In contrast, natality of Steller sea lions estimated in our study is substantially higher than the recently published estimate for the 2000s of 43% (Figure 1) which was inferred from a population dynamic model [13]. Our estimate of natality at Chiswell Island may be considered normal and indicative of a stable or increasing population, whereas the inferential model estimate [13] suggests a population that is still under stress, nutritional or otherwise. Notwithstanding variation in survival, natality rates of 60% to 75% have been generally associated with stable or increasing populations of pinnipeds [20]–[22], whereas rates of 55% or lower have been associated with declining populations and related to the adverse effects of density dependant factors or food stress [8], [23].

There are at least two possible reasons for the large discrepancy between the two estimates which we will examine briefly in turn. One is that the population status and natality trends in Kenai Fjords are not representative of the greater GOA. The other is that the methods for calculating natality were very different between the two studies making comparisons difficult; i.e. ours is a direct estimate, whereas the other [13] is a hypothetical value based on an inferential model.

**Figure 3:**
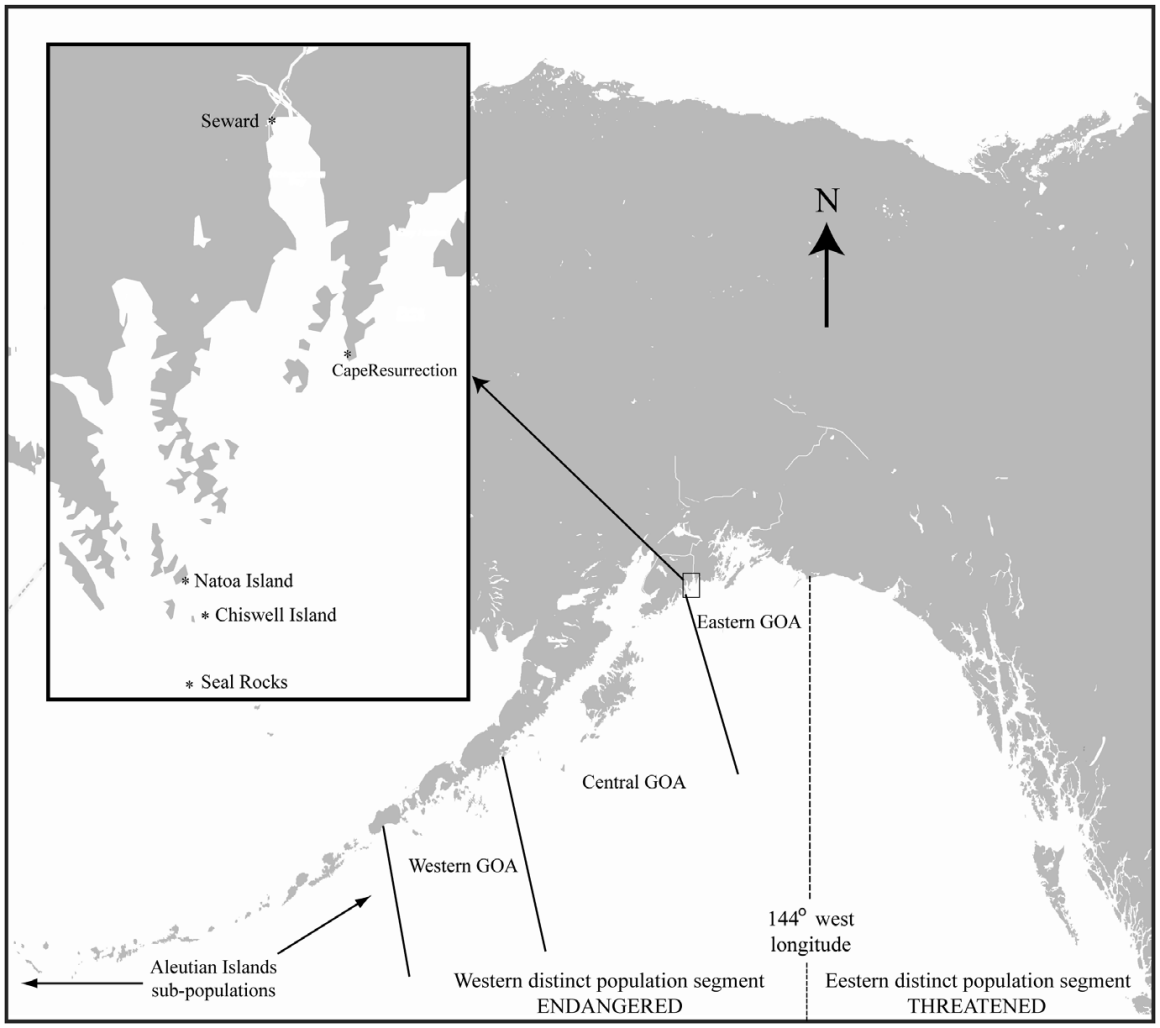
Location of the Chiswell Island Steller sea lion rookery and remotely monitored haulouts in Kenai Fjords, Gulf of Alaska.

The inferential population dynamic model [13] was based on data from only the central GOA (Figure 3), and was assumed to represent a major portion of the WDPS of Steller sea lions. Population trajectories within the WDPS vary widely with location [12] and we do not assume that our estimate of natality in Kenai Fjords is necessarily representative of natality throughout the entire western range of these animals. However, the evidence presented below suggests that our findings may be representative of the eastern and central GOA (Chiswell Island lies in the transition zone between these somewhat arbitrary regions; Figure 3).

Steller sea lions in the eastern GOA, which includes Chiswell Island, have experienced a 35% increase in their population over the period 2004–2008, while those in the central GOA increased by only 10% over that period [24], although it is argued that the large growth in numbers in the eastern GOA was due to a seasonal influx of animals from southeastern Alaska (the eastern distinct population segment) [24]. The increasing population trends of resident animals in the eastern GOA, therefore, are more equivalent to those in the central GOA. Furthermore, the ratio of adults and juveniles to pups counted in aerial censuses in the 2000s at Chiswell Island (median  = 1.64) is the same as at other rookeries in both the central and eastern GOA (median  = 1.71; Mann-Whitney U = 46.00, P = 0.296; based on data in [12]). An additional similarity between the Chiswell Island rookery and other GOA rookeries is that measurements of maternal care are excellent at Chiswell Island [25] and are comparable to maternal care at other rookeries in the central GOA [26], suggesting prey is readily available across this broad area. With similar trends in behavior, population trajectories, and observed ratios of age classes throughout these regions, we find no reason to suspect that natality of sea lions in this study is unusually high compared to sea lions elsewhere in the GOA.

The previously published estimates of natality were based on long-term census counts of adults, juveniles, and pups across a broad range of the GOA [13]. However, the data suffer from several biases including, but not limited to, confounding influences of neonatal mortality, temporary immigration, and changing female sightability. In order to estimate natality from counts of adult and pup Steller sea lions, and make them comparable to the pre-decline estimate, accurately determining neonatal survival is necessary to account for pups lost prior to the time of census. Overestimates of pup survival would reduce the calculated number of pups actually born, thereby reducing apparent natality. The authors of the population dynamic model [13] applied a survival correction to their life history matrices of 0.949 to account for neonatal mortality and assumed that it was constant over time. That correction factor was derived from counts of live and dead pups found on rookeries in the 1970s. However, the leading cause of mortality in young pups results from being washed away in high surf conditions with survival to three weeks of age estimated to be much lower in dedicated studies (0.679 [27] and 0.896 [28]). Although there is currently no evidence that there was a significant change in Steller sea lion pup mortality over time [29], there can be significant variation in pup mortality between years [27], [28] and a high estimate of survival, assumed to be constant, would have decreased all estimates of natality over the periods studied.

Immigration, whether temporary or permanent, of animals from the growing southeastern Alaska population in recent years [12] will also skew census-based estimates of natality lower because animals that are not part of the breeding population may be counted as breeders. It is not known how much temporary immigration might have affected estimates of declining natality in the 2000s but some effect is probable.

It is also likely that female sightability in the GOA has changed systematically between ocean climate regimes in recent decades causing the appearance of reduced natality based on estimates of the proportion of animals hauled out. That is, if more females were hauled out during surveys in recent years compared to earlier years, then there would have been an appearance of reduced natality based on relative proportions of females to pups. The authors of the population dynamic model [13] assumed female sightability was constant over time and suggested that an increase of about 40% in the number of females observed would be necessary to counter the estimated decline in natality. There is no direct evidence of a long-term, increasing trend in female sightability but some compelling indirect evidence is explained as follows.

**Figure 4:**
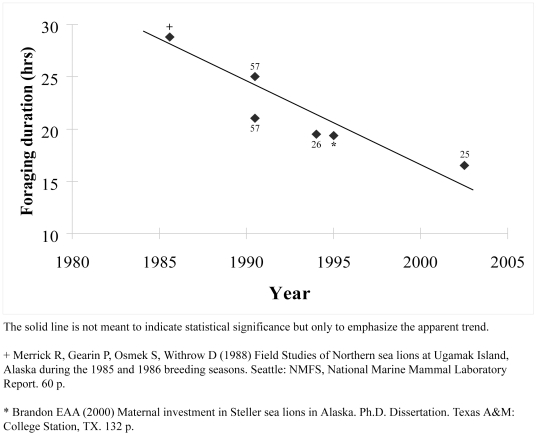
Steller sea lion foraging trip durations at rookeries between the mid-1980s and 2000s.

The availability of some important prey for Steller sea lions was probably reduced during the 1980s [6], [8], [15], [29]. Therefore, females would have spent more time foraging at sea to adjust for prey deficiency during that time. Such behavioral changes associated with food limitation in otariids make sense and have been observed in other studies [30]–[32]. As sea lion populations continued to decline through the 1990s, changing ocean climate regimes probably led to improved forage availability [15], [16], which resulted in a systematic reduction in foraging durations by adult females (Figure 4). Shorter foraging periods would effectively cause an apparent decline in natality rates when simply counting ratios of adults to pups because more adults would be counted in relation to the number of pups in later years. There is no good information on perinatal periods (the time females spend on shore between giving birth and their next foraging trip to sea) in the 1980s, but perinatal periods are also greatly affected by nutritional limitation in the same way as foraging trip durations [30] and would therefore exacerbate the effect of female sightability across changing prey regimes. Hence, it may not be possible to accurately determine changes in pinniped vital rates based on census data without a complete understanding of how environmental factors affect sightability of different age-classes throughout a region over extended periods.

Further evidence of a healthy population in our study is indicated by lack of a cost of reproduction. Female reproduction is normally believed to carry costs in terms of a reduced likelihood of survival and/or future reproductive potential [33]. This effect has been shown in some studies of pinnipeds [34], [35] including Steller sea lions during the 1980s when pregnancy was negatively correlated with lactation status [8]. We found the opposite effect in this study with reproduction being positively correlated with survival (though not significantly so) and future reproduction, suggesting variation in overall fitness between individuals rather than a reproductive effect on fitness. Similar findings were reported for subantarctic fur seals (*Arctocephalus tropicalis)* [36], and strong evidence for an effect of individual quality on reproductive success has been seen in other large mammals [37]. Such variable reproductive strategies between fit and unfit individuals are most evident when resources are plentiful. Alternatively, when food resources are more limited, reproductive costs on future reproduction are more evident [37]. This provides additional evidence that lowered fitness and associated costs of reproduction in Steller sea lions during the 1980s were consistent with resource limitation [8], whereas the findings in this study of no cost of reproduction during the 2000s suggest that resources are more plentiful. In recent decades, most researchers agree that prey limitation is not a problem for Steller sea lions in the GOA [4], [38].

This study found that natality in Steller sea lions at Chiswell Island is at a level similar to that before the population decline, and evidence presented above suggests that the animals in Kenai Fjords could be representative of those across the eastern and central GOA, but not necessarily further afield. Population losses during the 1980s are thought by some researchers to have been caused by nutritional stress resulting from the ocean climate regime shift in the mid-1970s [6], [29], [39], although others disagree [5], [7], [40]. Indeed, there is good evidence that juvenile survival and recruitment was reduced by predation [5], [41] and/or food limitation [19]. Nevertheless, it is plausible that decreased natality in the 1980s compared to the 1970s was caused by nutritional limitation during that period and that it may explain some of the population decline [6], [8]. In more recent years, studies of juvenile health and maternal care provide no evidence of nutritional limitation in this species [25], [27], [38], [42]. Disease, parasitism, and contaminants could adversely affect reproduction [8], but research has not shown significant trends over time or major problems in the current decade [43], [44]. Other explanations for population losses, such as predation and fisheries related mortalities, could play a major role in adult and juvenile survival but probably have less of an effect on natality, although exposure to predation risk does increase levels of stress in female Steller sea lions [45] and can decrease natality in other species of large mammals [46].

Given the evidence presented here, we suggest that the apparent long-term decline in GOA Steller sea lion natality as inferred elsewhere [13] is probably due to an artifact of increasing female sightability as resources became more abundant from the 1980s to present. Other factors such as neonatal mortality and immigration may have also affected those inferential estimates of natality. Direct estimates during the 1980s provided sufficient evidence of a reduction in natality only during that time period [8], but those findings do not necessarily exclude the additional, possibly more important role of top-down effects of predation in the collapse of WDPS Steller sea lions [5]. Our result suggests that natality of Steller sea lions in the 2000s is similar to that before the population decline (1970s) and is consistent with natality found in stable or increasing pinniped populations.

The contrasting results presented here and by the authors of the population dynamic model [13] have major implications on our understanding of factors at play in the GOA ecosystem that affect Steller sea lion populations, and by association populations of harbor seals (*Phoca vitulina*) and sea otters (*Enhydra lutris*) that also collapsed during the same era in the same region [5], [47], [48]. There is no evidence of a nutritional mechanism that might have driven natality of Steller sea lions down to such a low level subsequent to the late 1980s as inferred by the population dynamic model [13], and low natality is opposite that which would be expected in an otherwise healthy population of animals. The different estimates also have important implications on management strategies that have been, and might be, enacted to help Steller sea lion populations recover. Resolving uncertainties that have arisen from the two approaches to estimate natality, i.e., whether there is a systematic difference between them, could easily be put to a direct test by applying them simultaneously at several rookeries in the GOA. Until then, attempts to explain the lack of recovery of the WDPS in the GOA should more fully explore alternative hypotheses to nutritional limitation, such as high predation mortality of juveniles as suggested by recent findings of Horning and Mellish [49].

## Materials and Methods

### Ethics Statement

This study meets all ethical standards based on an approved Animal Care and Use Committee permit and National Marine Fisheries Service permits to conduct research on Endangered Steller sea lions.

### Study Site and Observational Methods

This study was conducted at the Steller sea lion rookery on Chiswell Island and nearby haulouts in Kenai Fjords (Figure 3) which lie within the range of the endangered WDPS. The pattern and magnitude of population decline at the rookery were similar to other rookeries in the central GOA—that is, abundance fell by 90% from 1,459 adults and 564 pups in 1956 [50] to approximately 90 adults and 50–80 pups in the 2000s [25].

Beginning in 1999, up to six remotely operated video cameras were used to monitor Steller sea lions (see [25] for details). Video images, which provided complete spatial coverage of the Chiswell Island rookery, were viewable and controllable in real-time from the Alaska SeaLife Center 65 km away. Cameras were also installed and monitored at nearby haulouts beginning in 2000 (Figure 3).

Most adult Steller sea lions can be individually identified by unique scars, fungal patches, and/or flipper patterns and longitudinal studies have been successfully conducted on animals identified by such means [25]–[27]. During the course of this study, female sea lions with unique markings were tracked and digital photos of those animals and their distinguishing marks were taken on a regular basis from all remotely-monitored sites in Kenai Fjords. A few breeding females were identified by flipper tags (n = 4) or brands (n = 2), and age was known only for those animals. Females that did not have at least two distinguishing marks and could not be reliably resighted from one year to the next were not used in this analysis. Although pictures and data for some females were collected as early as 1999, they were not considered during1999–2002 in the analysis of natality rates because of more focused sighting effort on those giving birth over those that did not in those years. All females with unique markings (an average of 68.9%±4.8% SE of the Chiswell Island female population in each year) were non-preferentially identified and tracked from 2003 onward whether or not they gave birth.

Observations each year began with the arrival of the first female on the rookery in mid- to late-May and included full census counts of all sea lions by age-class (male/female adult, juvenile, yearling, and pup) on the rookery throughout the breeding season. Census counts were made at approximately 1100 h and 1900 h, and hour-long scan sampling for identifiable females and their pups was done four to ten times daily from 0600 h to 2200 h; earlier and later hours were added around the summer solstice when light levels were sufficient for viewing sea lions. After 10 August, observations were recorded from approximately sunrise to sunset as diminishing daylight allowed. Events such as births and deaths were opportunistically recorded as they occurred or within 4 hr of their known occurrence [28]. Births that happened overnight were recorded the following morning as having occurred at the half-way point of non-observation hours.

Steller sea lion mothers in the WDPS will normally remain with their newborn pups for 8 to 12 days following parturition [25], [26]. Given the duration and detail of observations in this study (frequent scans and complete spatial coverage of the rookery), it was highly unlikely that any births went unnoticed. Identified females were considered for this analysis if they were present on the Chiswell Island rookery during the pupping and breeding season from 15 June until 15 July. Females that gave birth earlier still had a definitive presence on the rookery during that time. That time period also included females that were present to copulate, and hence had a presumed intention to breed at this rookery, but excluded some females that hauled out briefly on Chiswell Island before leaving to potentially pup elsewhere.

Typically, females that give birth to stillborns should not be considered productive. However, all recent estimates of natality in Steller sea lions are compared to natality in the presumed healthy population during the 1970s and declining population in the 1980s. Those earlier estimates were based on late-term pregnancies and could not account for stillbirths [8]. Therefore, full-term stillbirths were included as births in this study to make the data comparable to those earlier standards, but this probably had little effect on the estimates of natality because fewer than 2% of pups born at Chiswell Island were stillborn [28]. Furthermore, the published standards for natality were only considered for reproductively mature females whose status was known by examination of ovaries [8], [14]. It was not possible to verify reproductive maturity in this study even when age was known. To reduce the chance of including pre-reproductive animals in our dataset, the first year of sighting of each apparently mature female of unknown age was removed whether or not she gave birth. Those that gave birth in their first year of observation were removed to avoid bias toward more fecund animals. Females of known age were included in this study beginning at 5 years of age to be consistent with the average age of sexual maturity at 4.6 yr [14], which would indicate that age of first pupping would be at about 5.6 yr.

In order to decrease sample bias toward more fecund females that may spend proportionally more time at a rookery, nearby haulouts were also monitored during the pupping season to account for females that may have spent more time at those locations. Females at haulouts were included in the analyses if they met the abovementioned sighting and maturity criteria unless they were accompanied by juveniles that were known to be born elsewhere (i.e., not at the Chiswell Island rookery). Many of the animals in the Chiswell Island population that were not giving birth on the rookery in any given year spent the summer elsewhere, presumably outside of the study area. Females that returned to the study area later in the year without a pup were classified as not giving birth in that particular year because of known breeding-site fidelity in this species [51] and to ensure a conservative approach to estimated natality.

### Data Analysis

Multi-state models [52] were constructed using the logit link function in Program MARK with the following parameters being estimated over 7 years of observation (2003–2009):

S*_i_* ^x^ = probability that a female in state x in year *i* survives until *i* +1.

P*_i_* ^x^ = probability that a female is resighted in year *i* in state x, given that it is present in the study area in year *i*.

ψ*_i_* ^xy^ = probability that a female in state x in year *i* is in state y at *i*+1, given that she survived from year *i* to *i* + 1.

States were recorded as “B”–observed birth or with pup, “N”–observed but did not give birth or not seen with pup, and “0”–not observed. Calculation of the proportion of females that were productive (natality) was performed using equation 2 in Nichols et al. [53] with corresponding estimates of variance.

The Cormack-Jolly-Seber (CJS) modeling approach was chosen over models that account explicitly for temporary emigration because CJS models required fewer assumptions and constraints in addition to providing sufficient parameter estimates for animals that show breeding site fidelity [35] as Steller sea lions do, especially after breeding has been established [51]. Sighting probabilities for the two strata (B and N) were retained in tested models to express breeding-site fidelity and differences in the ability to detect those states.

We compared models with Akaike's Information Criteria (AIC)[54], corrected for small sample bias (AIC_c_)[55] with additional comparisons for nested models using likelihood ratio tests (LRT). The general, fully time and state dependant model was initially tested for goodness-of-fit (GOF) with program U-CARE 2.2 [56] and the estimated overdispersion coefficient (ĉ) was used to adjust model results and convert AIC_c_ values to quasi-AIC_c_ (QAIC_c_) values. QAIC_c_ weights, calculated from model differences in QAIC_c_ values (ΔQAIC_c_), indicated relative support for the various models.

Finally, we examined indicators of potential costs to giving birth in regard to survival and state transitions. Cost is suggested if birthing in one year is associated with a significant reduction in survival probability for the following year. Birthing in one year may also cause a reduction in the probability of birthing in the next year, as was indicated for Steller sea lions in the 1980s [8]. An effect of birthing on subsequent birthing is suggested if transitions from not birthing to birthing (ψ^nb^) were greater than transitions from birthing to birthing (ψ^bb^)[35].
